# Evaluating the effect of arachidonic acid and eicosapentaenoic acid on induction of adipogenesis in human adipose-derived stem cells 

**DOI:** 10.22038/ijbms.2020.41557.9819

**Published:** 2020-08

**Authors:** Rezvan Mostoli, Farjam Goudarzi, Adel Mohammadalipour, Iraj Khodadadi, Mohammad Taghi Goodarzi

**Affiliations:** 1 Department of Clinical Biochemistry, School of Medicine, Hamadan University of Medical Sciences, Hamadan, Iran; 2 Regenerative Medicine Research Center, Kermanshah University of Medical Sciences, Kermanshah, Iran; 3 Department of Clinical Biochemistry, Faculty of Pharmacy and Pharmaceutical Sciences, Isfahan University of Medical Sciences, Isfahan, Iran

**Keywords:** Adipogenesis, Arachidonic acid, Eicosapentaenoic acid, GLUT4, Human adipose-derived - stem cell, PPARγ2

## Abstract

**Objective(s):**

Adipose tissue is one of the most important endocrine organs that liberates many metabolic mediators such as hormones, cytokines, and chemokines. Different types of fatty acids have key roles in adipogenesis. The aim of this study was to evaluate the effects of two essential fatty acids, including Arachidonic acid (AA) and Eicosapentaenoic acid (EPA), on the process of adipogenicity in human Adipose-Derived Stem Cells (hADSCs).

**Materials and Methods:**

After immunophenotyping of hADSCs by flowcytometry, they were differentiated into adipocytes and simultaneously exposed to 30 μM and 60 μM of AA and 25 μM and 50 μM of EPA. Further, along with the MTS assay, the activity of glycalaldehyde-3-phosphate dehydrogenase (GAPDH) was also measured. In addition, expression of lipid markers including peroxisome proliferator-activated receptor γ2 (PPARγ2) and glucose transporter 4 (GLUT4) was evaluated, and the neutral lipid contents were determined using Oil red O staining.

**Results:**

MTS evaluation showed a significant decrease in proliferation in all treatment groups compared to the control group. Based on oil red O staining, fat droplets in the AA treatment groups were higher than in controls. The expression of PPARγ2 and GLUT4 genes and proteins increased in almost all AA and EPA groups compared to control. In addition, GAPDH activity was higher in AA groups than in the control group. In general, while different concentrations of EPA did not increase the adipogenic process compared to the control group, stimulation of differentiation to adipocytes was largely determined by the AA.

**Conclusion:**

The result indicates a positive effect of omega-6 versus omega-3 in stimulating the pathways of adipogenesis.

## Introduction

Adipose tissue is known as an energy storage source and one of the most important endocrine organs that liberates many metabolic mediators. These metabolites include hormones, cytokines, chemokines, and other immune-related factors. Therefore, in the process of obesity, overproduction of these compounds due to hypertrophic (increased volume of adipocytes) and hyperplasia (increased number of adipocytes) of adipocytes leads to poor cellular messaging throughout the body. As a result, its outcome includes chronic inflammation, vascular and metabolic changes, hypoxia, decreased insulin sensitivity, and increased inflammation of the tissues during cellular autophagy and apoptosis (1). Adipocyte hyperplasia that is related to the severity of obesity arises from adipogenic differentiation of neighbors multipotent mesenchymal stem cells (MSCs) (2). This differentiation happens through the effect of regulatory factors such as C/EBPβ (CCAAT/enhancer binding protein β), C/EBPα (CCAAT/enhancer binding protein α) and peroxisome proliferator-activated receptor γ (PPARγ) (3). Furthermore, the lipid deposition related to adipocyte differentiation is happening as a result of the contribution of glyceraldehyde 3-phosphate dehydrogenase (GAPDH) and glycerol 3-phosphate dehydrogenase (G3PDH) activities. 

Given the importance of fat differentiation in the severity of obesity, and the role of fatty acids as signal transduction molecules in this process, researchers have focused on the key role of different types of fatty acids in differentiation. Administration of natural fatty acids or their systemic agonists (thiazolidinediones) has been observed to induce adipogenesis by activating PPARγ (4, 5). Interestingly, while palmitic acid supplementation showed a strong stimulatory effect on differentiation, conjugated linoleic acid suppressed differentiation and reduced adipocyte markers such as fatty acid binding protein (aP2) or PPARγ in 3T3-L1 adipocytes (6, 7).

According to scientific evidence, administration of polyunsaturated fatty acids (PUFA) increases lipid oxidation and inhibits adipogenesis by regulating signaling molecules engaged in fatty acid transportation (8). Similarly, docosahexaenoic acid (DHA, C22:6), (n-3) PUFA, like fish oil that is produced from its precursor, and eicosapentaenoic acid (EPA) reduced preadipocyte differentiation and its subsequent lipid accumulation (9). On the other hand, administration of n-3 PUFA contributes to an increase in the expression of PPARγ, adipogenesis, lipid droplets, and the spread of adipose tissue during a positive energy balance (10). In addition to n-3 PUFA, arachidonic acid (AA) as the n-6 PUFA, is another fatty acid supplementation that is abundant in the Western diet and is important as a signaling molecule (11). Eicosanoids as the most important groups of bioactive lipid mediators are derived from AA and modulate multiple mechanisms in relation to adipocyte metabolism (12). AA can inhibit adipocyte differentiation, via generation of prostaglandin F2α (PGF_2α_) through cyclooxygenases (COXs) and regulation of adipocyte protein 2 (aP2) (11). Furthermore, while it accelerates early differentiation by lipooxygenase (LOX), it could hamper adipocyte differentiation by COX in long-term treatment. In comparison between n-6 and n-3 fatty acid supplementation in the adipogenesis process, it has been recently documented that increase the ratio of n-6/n-3 in human milk is considered one of the potential markers of childhood obesity (13). Therefore, according to the abovementioned, no clear panel in relation to precise n-6 and n-3 compounds has been shown on the adipogenesis process. Therefore, in this study, the effect of these two agents (AA and EPA) was investigated in the cellular level of adipocyte differentiation. 

## Materials and Methods

***Isolation and preparation of adipose-derived mesenchymal stem cells***

The isolation of MSCs was carried out by employing the discarded lipoaspirates. The ethical committee of Hamadan University of Medical Sciences approved the protocol of this study. Accordingly, the isolated cells were prepared from healthy women (n=4) aged between 25 to 35 years old. After washing of fresh lipoaspirate with PBS (0.15 M, pH = 7.45) and penicillin/streptomycin (5%) (Kiazist Life Sciences, Iran), the collagen digestion was performed for one hour. Collagenase type 1 (Thermo Fisher Scientific, USA) solved in PBS (1 mg/ml) was used following multiple shaking at 37 °C in a humid atmosphere containing 5% CO_2_. After that, the collagenase solution was neutralized by adding the same volume of complete growth medium containing DMEM-f12 and 10% FBS (Kiazist Life Sciences, Iran). In the following steps, the blood cells were separated with the help of RBC lysis buffer (pH=7.3) and centrifugation. The attached cells were re-suspended by complete growth medium and transferred to the T-25 flask. The morphological shapes and specified CD markers were studied to authenticate the specific MSCs. 

***Immunophenotyping of ADSCs***

To confirm the MSCs by flowcytometry, the isolated Adipose-Derived cells (ADSCs passage number>3, about 1×10^6^ cells) were trypsinized, fixed with 4% paraformaldehyde (Sigma-Aldrich, UK) and transferred to a FACS tube. The fluorochrome-conjugated antibody against positive markers of CD90, CD73, and CD44 (BD Biosciences, USA) and negative ones including CD34 and CD45 (Abcam, UK) were determined by flowcytometry (Partec, Germany). Rabbit polyclonal IgG was used as isotype controls (BD Biosciences, San Jose, USA). 

***Adipogenic differentiation of ADSCs and their treatment***

Cultured ADSCs (passage number>3, about 1×10^6^ cells) were induced to differentiate into adipocytes using differentiation medium. The medium contained 1 µM dexamethasone, 10 µg/ml insulin, 100 µM indomethacin and 0.5 mM of 3-isobutyl-1-methylxanthine (IBMX) (All from Sigma-Aldrich, USA). The prepared cells were also treated with 25 µM and 50 µM of EPA (EPA25 and EPA50) and 30 µM and 60 µM of AA (AA30 and AA60) (Sigma-Aldrich, USA) from the beginning of differentiation. These fatty acids were bound to 1% albumin for 15 min at 37 ˚C. Oil red O lipid staining was performed to illustrate lipid droplets. The staining was performed according to our previous study (14). Briefly, the Oil Red O working solution (0.3% Oil red O dissolved in 0.18% isopropanol) was added to the fixed cells (in 10% formalin) and incubated for 10 min at room temperature. The Hematoxylin dye was added immediately in the next step. After one minute, the cells were washed and examined and photographed using an inverted light microscope (Nikon, eclipse ts100, Japan). Oil Red O pixel counting was performed by NIH ImageJ software (Version 1.8.0_112). 

***ADSCs proliferation assay***

ADSCs proliferation assay was perfumed using MTS (3-(4,5-dimethylthiazol-2-yl)-5-(3-carboxymethoxyphenyl-2-(4-sulfophenyl)-2H-tetrazolium) assay kit according to the manufacturer’s instruction (Abcam, UK). For this purpose, the differentiation medium was replaced by complete growth medium (200 µl) for each well of 96 well plates containing cultured cells. After incubation of the cells in this culture medium for 24 hours at 37 °C in a humid atmosphere containing 5% CO_2_, the MTS reagent (20 μl/well) was added to each well and the plate was incubated at the same mentioned condition. Finally, after four hours of incubation, the absorbance was determined at 490 nm in a Synergy H1 Multi-Mode Reader (Bio-Tek Instruments, USA). The viability is directly related to the absorbance of each group.

***GAPDH activity assay***

The rate of differentiation was also evaluated by the measurement of the activity of GAPDH. Therefore, using centrifugation at 14000×g, the cell lysate was prepared. Then, the GAPDH activity was assayed according to GAPDH assay kit (abcam, Cambridge, UK) protocol in 500 µl of lysate. 

***Real-time PCR***

To evaluate the expression of PPARγ2 and glucose transporter 4 (GLUT4) genes by Real-Time PCR, cells that were cultured in a T25 flask were used. For this purpose, extraction of RNA, treated with DNase I and cDNA synthesis were performed based on the previous study (15). Briefly, TRizol (Thermo Fisher Scientific, Budapest, Hungary) and NanoDrop (Onec UV-Vis Spectrophotometer Thermo Scientific) were used for RNA extraction and subsequent qualification of the extracted RNA. Revert Aid First Strand cDNA Synthesis Kit (Thermo Fisher Scientific, USA) was also used in the next step for cDNA synthesis. The primer pairs as shown in Table 1 were implemented. Real-Time PCR (qRT-PCR) was performed using SYBR green qPCR mastermix via LightCycler® 96 System, (Roche, Germany). The relative gene expression was finally measured using the comparative cycle threshold (Cq) 2^–(∆∆Cq)^ method versus RPII gene expression as a house-keeping gene. 

***Western blot***

Briefly, the cells cultured in 60 mm dishes were detached by scraping and then lysed by radio-immunoprecipitation assay (RIPA) buffer (Santa Cruz Biotechnology, USA) containing protease inhibitors (Sigma Aldrich, MO). The total protein concentration was determined by BCA Protein Assay kit (Kiazist Life Sciences, Iran) and was heated for denaturation at 100 °C for 5 min. The electrophoretic separation of the protein (50 µg protein from each group) was carried out using sodium dodecyl sulfate-polyacrylamide gel electrophoresis (SDS-PAGE) in 10% gel under reducing conditions. After that, the separated proteins were transferred onto nitrocellulose membrane using semi-dry transfer membrane systems (Cleaver scientific, Warwickshire, United Kingdom). The membranes were then washed in TBS buffer, blocked by 3% BSA in TBST buffer for 2 hr, incubated with primary antibodies overnight at 4 ^o^C and secondary antibody for one hour. The signal was detected with an enzyme-linked chemiluminescence detection system (Kiazist Life Sciences, Iran). The primary antibodies (diluted in TBST buffer) were as follow: goat polyclonal anti-PPARγ_2_ (1:500), anti-GLUT4 (1:1000) and anti-cofilin (1:500) antibody, and secondary antibody was HRP-conjugated anti-goat antibody (diluted in TBST buffer; 1:10000), (All Santa Cruz Biotechnology, USA). The TBS buffer included 20 mM Tris HCl, and 500 mM NaCl (pH=7.4), and the TBST buffer included 20 mM Tris HCl, 500 mM NaCl, and 0.5% Tween 20 (pH=7.4). 

***Statistical analysis***

Cells obtained from four donors were examined as triplicate in two independent experiments. The results were analyzed using one-way ANOVA followed by *post hoc* Tukey’s test. All results were presented as mean±SEM of three independent experiments and *P*-values less than 0.05 were considered as statistically significant. The plot was designed using GraphPad Prism version 6.0 (GraphPad Software, San Diego-USA). 

## Results

***Human ADSCs immunophenotyping characterization***

**Figure 1:**
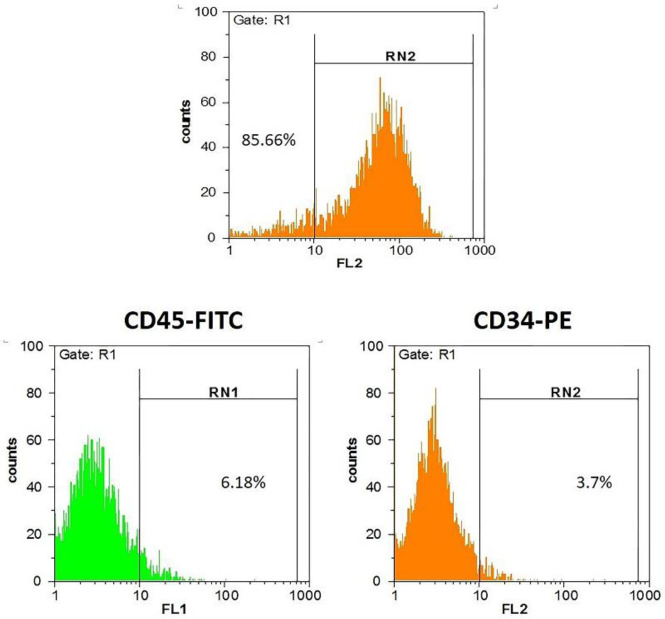
Flow cytometric analysis of exposed CD markers at 3^rd^ passage. As it observed, the cells express low level of CD34 and CD45 (3.7% and 6.18% respectively), and high level of CD44, CD73, and CD90 (90.22%, 95.31%, and 85.66%). FITC: Fluorescein isothiocyanate. PE: phycoerythrin

The both positive (CD73, CD105, and CD29) and negative (CD34 and CD45) markers of MSCs were analyzed by flowcytometry. Accordingly, population of MSCs phenotype were confirmed with high expression of CD73, 95.31%; CD105, 90.22% and CD29, 85.66% and low expression of CD34, 3.7% and CD45, 6.18% (Figure 1). 

***MTS assay***

**Figure 2:**
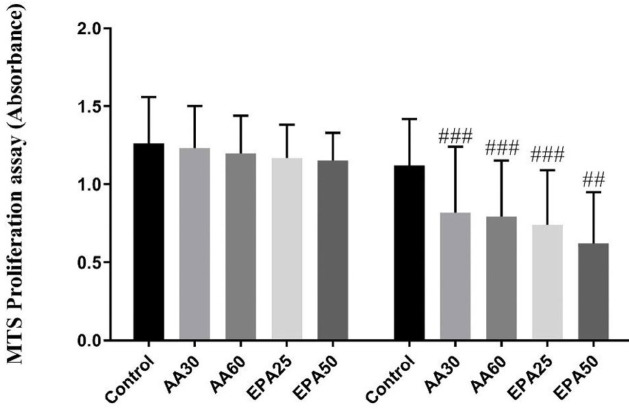
The MTS assay of cells in day 7 and 14 of differentiation. While the cell growth and viability showed no significant changes between all groups on 7^th^ day of differentiation, the significant lower viability and growth are observed in treated groups versus control group on 14^th^ day of differentiation. ## *P*<0.01 versus control and ### *P*<0.05 versus control. Results are presented as mean±SEM of three independent experiments

As is shown in Figure 2, the growth and proliferation of cells did not significantly increase until the seventh day of the induction period. With the continuation of the induction and enhancement of the adipogenic process, the growth rate of the cells was decreased. Cell growth of AA and EPA treated groups on day 14 was significantly lower than the control group, and the growth of these cells in the treated group with EPA50 was significantly lower than the rest of the groups (*P*<0.01).

***Oil red O determination of ADSCs differentiation***

**Figure 3:**
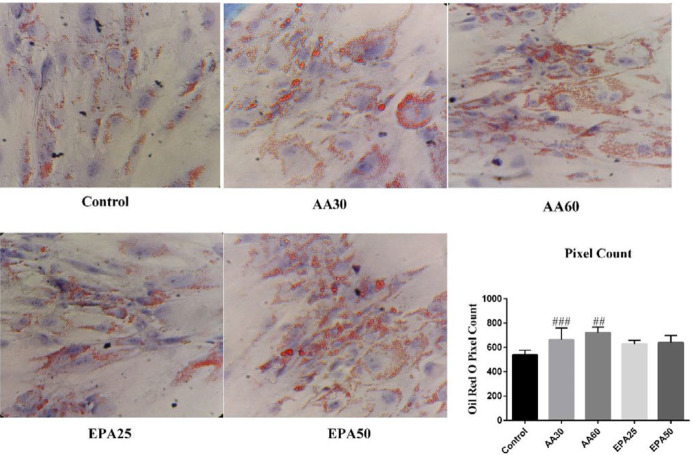
Oil red O staining of the cells of control, AA30, AA60, EPA25 and EPA50 groups. The lipid droplets stained with red color in counterstained blue color are observed. The higher increase in fat accumulation and lipid droplet was seen in AA30 and AA60 group compared with control and EPA groups. Magnification of 100X at room temperature ## *P*<0.01 versus control and ### *P*<0.05 versus control

AA: Arachidonic acid; EPA: eicosapentaenoic acid

After 14 days of induction, the morphology of the control group was changed from the spindle shape of ADSCs to oval shape of adipocyte. Moreover, a higher increase in fat accumulation and lipid droplet was observed in all treatment groups, which was significant in the AA groups compared to the control (*P*<0.025 *v.s* AA30 and *P* =0.001 *v.s* AA60) (Figure 3). So, the AA and EPA groups were differentiated to adipocyte almost more than the control group. Therefore, lipid droplets were observed in all groups with varying degrees. This suggests that the pathway for the induction of cells to adipocyte has occurred in all groups. The similar rate of adipogenicity between AA and EPA groups were found in the early days of differentiation. Subsequently, with the daily observation of cells during the fourteen days of induction, the rate of adipogenicity in different groups of AA, especially at higher concentrations, seemed to be more pronounced. 

***GAPDH activity***

**Figure 4:**
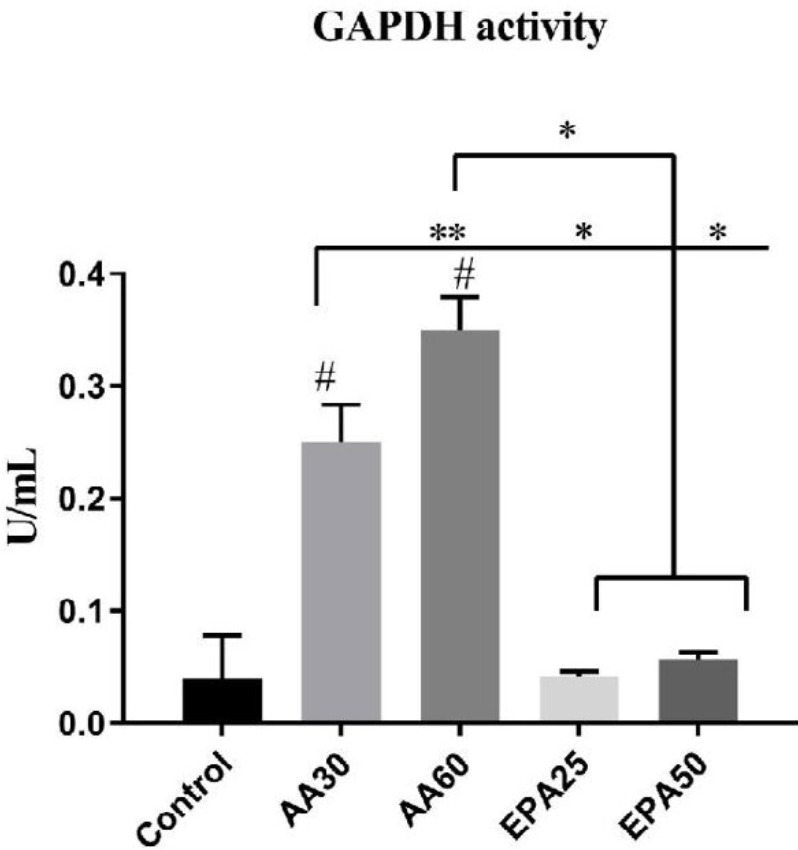
GAPDH activity assay in control, AA and EPA groups. Compared with control group, significant higher GAPDH activity was found in AA30 and AA60 groups. In AA60 group significant higher GAPDH activity was observed compared with AA30 group. Data are shown as mean±SD. # *P*<0.001 versus control. *P<0.001 and **P<0.01 (between treatment groups). Results are presented as mean±SEM of three independent experiments

GAPDH: glycalaldehyde-3-phosphate dehydrogenase; AA: Arachidonic acid; EPA: eicosapentaenoic acid GLUT4: glucose transporter 4

The results of enzyme activity assay are shown in Figure 4. As indicated in the diagram, while no significant changes in EPA25 and EPA50 groups were observed compared to the control group, the enzyme activity in the AA30 and AA60 groups was significantly higher than the control group (*P*<0.001). Interestingly, the higher concentration of AA (60 µM) showed a significantly higher GAPDH activity as compared to lower AA treatment (30 µM) (*P*=0.005) and cells treated with EPA25 and EPA50 (*P* <0.001). This indicates that the concentrations of the omega-6 were effective in increasing the activity of this enzyme and that the omega-3 supplementation did not have any significant effect. 

***Gene expression analysis***

**Figure 5:**
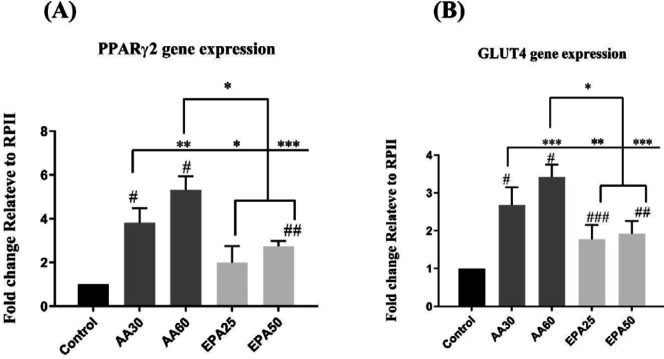
Gene expression of PPARγ2 (A) and GLUT4 (B) in AA and EPA groups. There was a significant increase in gene expression of PPARγ2 in AA30, AA60 and EPA50 groups. Gene expression of PPARγ2 in AA60 group was significantly higher than in EPA25 and EPA50 groups (*P*<0.05). Gene expression of GLUT4 in all AA and EPA groups is significantly higher than control group (*P*<0.05). Gene expression of GLUT4 in AA60 group is significantly higher than EPA25 and EPA50 groups (*P*<0.05). Data are shown as mean±SD. # *P*<0.001 versus control. ## *P*<0.01 versus control. ###*P*<0.05 versus control. *P<0.001, **P<0.01, and ***P<0.05 (comparing between treatment groups). Results are presented as mean±SEM of three independent experiments

As it is shown in Figure 5, the fold change expression of PPARγ_2_, as the main marker of adipogenesis, was significantly higher in all intervention groups (omega-3 and omega-6) than control groups (except the groups treated with 25 µM of EPA) (*P*<0.001 in AA30 and AA 60 versus control and *P*=0.001 in EPA50 versus control). In comparison between AA and EPA treated groups, significant higher PPARγ_2_ expression was observed in the AA60 group compared to both EPA25 and EPA50 groups (*P*<0.001). Treating cells with a lower concentration of AA (AA30) also led to the expression of significantly more PPARγ_2_ in these groups than different concentrations of EPA (*P*<0.001 *vs* EPA25 and *P*=0.038 *vs* EPA50). Subsequently, the expression of GLUT4 as another indicator of adipogenesis showed a significant increase in all treatment groups than the control group (*P*<0.001 in AA30 and AA60 *vs* control, *P*=0.016 in EPA25 and *P*=0.004 in EPA50 *vs* control). This issue shows the effect of omega-3 and omega-6 fatty acids on the induction of adipogenesis and the expression of the lipid metabolite marker, GLUT4. Additionally, treatment with AA60 and AA30 showed a significant increase in gene expression of GLUT4 compared to treatment with EPA25 and EPA50 (*P*<0.001 in the AA60 *vs* EPA25 and EPA50 and *P*= 0.004 in the AA30 *vs* EPA25 and *P*= 0.018 *vs* EPA50). Interestingly, the increase in EPA concentration did not show a significant change in the expression of GLUT4 gene, but higher AA concentrations than the lower concentration of this fatty acid significantly increased the expression of GLUT4 (*P*=0.024). 

***Protein expression of GLUT4 and PPARγ2 are concurrent with their gene expression***

**Figure 6:**
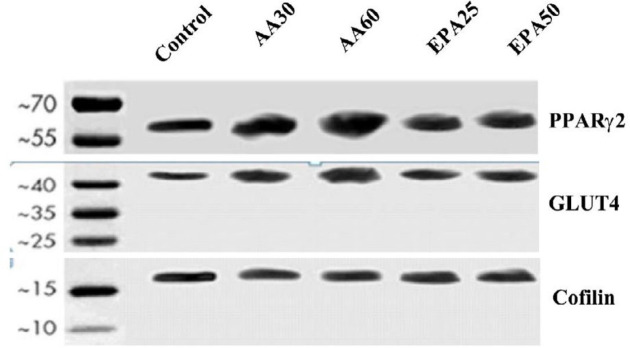
Protein expression of PPARγ2 and GLUT4 in AA and EPA groups by western blot. There was a higher protein level of PPARγ2 and GLUT4 in AA60 group versus control and the other treatment groups. Cofilin was used as a reference protein

Protein expression of PPARγ_2_ was higher in both AA (AA30 and AA60) treated groups than those of the control group and EPA treated groups (EPA25 and EPA50), (Figure 6). Similar results were observed for GLUT4 gene expression; however, there was no significant difference in the level of GLUT4 protein between two EPA groups (EPA25 and EPA50). The level of proteins was consistent with the results of the gene expression, indicating that the expression of genes has led to the protein stage.

**Table 1:** Primer sequences used in this study for gene expression analysis

**Gene Name**	**Primer Sequence**	**Product size (bp)**	**Ta**	**Accession Number**
**PPARγ**_2_	**Forward **CTATTGACCCAGAAAGCGAT **Reverse **CGTAATGTGGAGTAGAAATGC	210	54	NM_005037
**GLUT4 **	**Forward **AGGATCGGTTCTTTCATCTTCGC **Reverse **GTTCCCCATCTTCGGAGCCTA	98	59	NM_001042
**RPII**	**Forward **GCACCATCAAGAGAGTCCAGT **Reverse **ATTTGATGCCACCCTCCGTCA	81	57	NM_000937

AA: Arachidonic acid; EPA: eicosapentaenoic acid; MTS: 3-(4,5-dimethylthiazol-2-yl)-5-(3-carboxymethoxyphenyl)-2-(4-sulfophenyl)-2H-tetrazolium

PPARγ2: peroxisome proliferator-activated receptor γ2; AA: Arachidonic acid; EPA: eicosapentaenoic acid; GLUT4: glucose transporter 4

PPARγ2: peroxisome proliferator-activated receptor γ2; AA: Arachidonic acid; EPA: eicosapentaenoic acid GLUT4: glucose transporter 4

## Discussion

Multiple pathways related to adipogenicity have been concerned recently. So, the differentiation of MSCs into adipocytes can be affected by a variety of endogenous and exogenous compounds. Among all the exogenous compounds, long-chain polyunsaturated fatty acids (LC-PUFA) are the most important, since some omega-6 (n-6) and omega-3 (n-3) PUFA are termed as “essential”. Thus, according to the necessity of these supplementations, their effects on adipogenesis have been considered. They can be converted to longer unsaturated fatty acids with different structure and function by common similar pathways. Moreover, they regulate different biological processes such as inflammation, differentiation, and tumorigenesis (16, 17). However, they may be involved in the different mechanism of cellular metabolism related to adipogenesis. Accordingly, the probability of life-threatening effects of the high ratio (>10:1) of omega-6/omega-3 in the Western diet has been considered (18). In confirmation, the adjusted production of lipid compounds and inflammatory cytokines was observed in animal models that consumed a high-ratio omega-6 / omega-3 ration. (19, 20). However, their contrasting effect on adipogenesis and the trend of obesity has also been expressed (4, 5, 10). Therefore, in order to clarify these effects, two different concentrations of AA and EPA were used.

In our study, a significant decrease in proliferation in cells treated with two concentrations of AA and EPA was observed. The adipocytes differentiation occurs in two important stages of mitotic clonal expansion (MCE) and adipogenesis, which leads to the expression of the inducible genes of the adipocytic phenotype. This process occurs within two days of induction. By inhibiting cell growth in the MCE stage, PUFA has been shown to block adipogenesis and begin the process of apoptosis or cell necrosis (9). In the present study, the growth arrest and decreased cell survival did not occur during the first 7 days of differentiation, but this trend was observed on the 14^th^ day of this process. Other studies also demonstrated the anti-proliferative effects of PUFA in many cell lines (21, 22). 

In order to show the adipogenicity rate after 14 days, the oil red O staining was performed that showed more adipogenesis in cells treated with AA30 and AA60 (omega 6) compared to those treated with EPA (omega-3). It should be noted that staining of the lipid droplets is only related to the later stages of adipogenesis, while differences in fat droplets and their number were observed during the 14 days of intervention. In early studies relating to the effect of DHA (as an EPA product) on 3T3-L1 cells, adipogenic inhibition and fat production were shown to increase with DHA concentration (9). Also in other studies, the role of linoleic acid in controlling adipogenesis was expressed (6, 7). This diversity of the results observed in our findings and other studies in relation to the effects of various types of omega-3 and omega-6 compounds express the complexity of the effects of different types of omega-3s and omega-6s on this process. Also, it has been reported that DHA (omega-3) has greater anti- glyceroneogenesis effect than EPA (omega-3) (23). Another study also suggested that AA induces adipogenesis, but DHA and EPA inhibit adipogenesis in MSCs (24). However, in another study, AA inhibited the differentiation of adipocytes by producing PGF2α and regulating protein 2 adipocyte (aP2) (11).

Subsequently, in our study, morphological changes in adipogenesis were also evident in the change of GAPDH activity as an end marker. However, cells treated with AA showed higher activity of the GAPDH enzyme, indicating a high level of adipogenicity in these cells. GAPDH is an intermediate enzyme in the pathway of glycolysis that stimulates the production of pyruvate and produces acetyl-co-carboxylase, which is essential for lipogenesis. Interestingly, the changes in GAPDH activity in the EPA group were significantly lower than AA group, so the treatment of cells with EPA did not change the activity of GAPDH compared to the control group. This adipogenic process was also evaluated at the gene and protein expression levels of the PPARγ2, which in the AA group was more than EPA. This higher GLUT4 gene and protein expression in AA-treated cells, compared to the types of EPA, indicated the need for glyceroneogenesis in these cells when differentiated into adipocytes. The positive effect of Omega-3 PUFA on the induction of GLUT4 expression was also observed in a previous study (25). DHA and EPA oxidize fatty acids by activating PPARα and can stimulate UCP-2 expression in adipose tissue (25). As a result, the types of omega-3s and omega-6s reduced the synthesis of fatty acids by inhibiting SREBP-1 in the liver (26). Therefore, it has been shown that the intervention in the process of differentiation and increase in the number of adipocytes occurs in at least two paths. One by inhibiting at the MCE stage and the latter by inhibiting lipid accumulation in adipocytes and inducing apoptosis. In our study, as noted, unlike the effect of AA in stimulating adipogenesis within 14 days, the effect of EPA on this process was either ineffective or had a negligible positive effect.

The importance of AA in inducing adipogenesis was also reported at concentrations of 20 µM and 40 µM (27). In the present study, other concentrations of 30 µM and 60 µM of AA have also stimulated adipogenesis, which in fact confirmed the previous conceptions associated with the effect of AA on adipogenesis. In another study, 10 µM of AA inhibited adipogenesis by increasing the cAMP-elevating agents during the MCE phase (28). Therefore, the effects of AA on adipogenesis appear to occur in the advanced phase of the adipogenic process. It has been argued that PUFA-rich diets express adipogenic specificity factors, C/EBPα, and PPARγ2, and induce adipogenic differentiation (29). However, this effect was lower in rodent diets in n-3 PUFA supplements than in n-6s (30). The important point in our study is the use of physiological concentrations that was consistent with the *in vivo* results of some studies [30] and contrary to other studies (8).

## Conclusion

AA has an agonistic effect on the expression of adipogenic genes and showed an important role in adipogenesis comparing to the omega-3 fatty acid effect. It is conferred that when the cell needs to activate the lipogenic pathways, the effect of AA results in the production of ligands that ultimately increases PPARγ and adipogenesis and metabolic markers such as GLUT4. However, EPA, as an omega-3 PUFA, had a minimal stimulatory effect on the adipogenesis process within 14 days. So, the differences in the concentration of fatty acids in this study with others, the type of fatty acids used, and the duration of adipogenesis produced different results. Therefore, the mechanisms of the effects of EPA and AA in adipogenesis need to be cleared in more detail. 
